# Supplementary material for: MetaboTools: A Comprehensive Toolbox for Analysis of Genome-Scale Metabolic Models
Source: Front Physiol. 2016 Aug 3;7:327. doi: 10.3389/fphys.2016.00327 (PMC4971542; doi:10.3389/fphys.2016.00327)
Supplement: Supplementary file 1 [file DataSheet1.pdf]

MetaboTools: A comprehensive toolbox for analysis of genome-scale metabolic models

Maike K. Aurich[1], Ronan M.T. Fleming[1], and Ines Thiele[1*]

[1]Luxembourg Centre for Systems Biomedicine, University of Luxembourg, Esch-sur-Alzette, Luxembourg.
[*]Corresponding author: Ines Thiele, Luxembourg Centre for Systems Biomedicine, University of Luxembourg, 7, avenue des Hauts-Fourneaux, L-4362 Esch-sur-Alzette, E-mail: ines.thiele@uni.lu.

Table of Contents

1 Supplementary material 1
1.1 The model structure in matlab 1
1.2 Infinite constraints 3
1.3 Conversion of the theoretical mass 4
1.4 Calculate cell dry weight 4
1.5 Generate metabolic fluxes 4
1.6 Additional on scaling of infinite bounds and defined constraints 4
1.7 Additional remarks on the integration of Gene Expression data 5
1.8 Additional remarks on "Sampling the solution space" 5
2 References 6

1 Supplementary material

1.1 The model structure in matlab

This section explains the Matlab structure of a metabolic model based on the structure of the human genome-scale reconstruction or Recon2 [1]. The content of the model can be queried on http://vmh.life (Figure S2). Updated versions of Recon 2 can be downloaded from the same database. The Recon 2 model contains numerous variables (Figure S1). These are vectors or matrices and specify, e.g., the reactions, the metabolites, or the connections between these components.

The model can be loaded into Matlab, e.g., by navigating the current folder to the location where the model is saved and dragging it into the Matlab command window. Once the model is loaded, it appears in the workspace. Double-click on the model structure in your work-space to see the structure as in Figure S1.

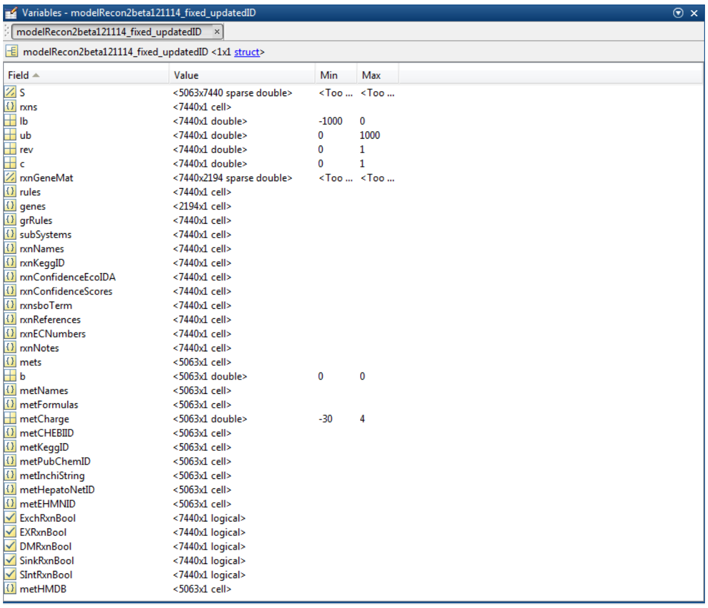

Variables - modelRecon2beta121114_fixed_updatedID

modelRecon2beta121114_fixed_updatedID

modelRecon2beta121114_fixed_updatedID <1x1 struct>

Field	Value	Min	Max
S	<5063x7440 sparse double>	<Too ...	<Too ...
rxns	<7440x1 cell>		
lb	<7440x1 double>	-1000	0
ub	<7440x1 double>	0	1000
rev	<7440x1 double>	0	1
c	<7440x1 double>	0	1
rxnGeneMat	<7440x2194 sparse double>	<Too ...	<Too ...
rules	<7440x1 cell>		
genes	<2194x1 cell>		
grRules	<7440x1 cell>		
subSystems	<7440x1 cell>		
rxnNames	<7440x1 cell>		
rxnKeggID	<7440x1 cell>		
rxnConfidenceEcoIDA	<7440x1 cell>		
rxnConfidenceScores	<7440x1 cell>		
rxnsboTerm	<7440x1 cell>		
rxnReferences	<7440x1 cell>		
rxnECNumbers	<7440x1 cell>		
rxnNotes	<7440x1 cell>		
mets	<5063x1 cell>		
b	<5063x1 double>	0	0
metNames	<5063x1 cell>		
metFormulas	<5063x1 cell>		
metCharge	<5063x1 double>	-30	4
metCHEBIID	<5063x1 cell>		
metKeggID	<5063x1 cell>		
metPubChemID	<5063x1 cell>		
metInchiString	<5063x1 cell>		
metHepatoNetID	<5063x1 cell>		
metEHMNID	<5063x1 cell>		
ExchRxnBool	<7440x1 logical>		
EXRxnBool	<7440x1 logical>		
DMRxnBool	<7440x1 logical>		
SinkRxnBool	<7440x1 logical>		
SIntRxnBool	<7440x1 logical>		
metHMDB	<5063x1 cell>		

Supplementary Figure 1: Variables in the human metabolic model.

The variables in the human metabolic model (Figure S1) can be divided into different categories: (1) matrices that connect metabolites with reactions or genes to reactions; (2) reaction variables that specify reactions, reaction identifiers, and other information relevant to the reactions; (3) metabolite variables that specify the metabolites, metabolite identifiers, and other information relevant to the metabolites; (4) variables for modeling (reactions); (5) boolean vectors to easily identify individual reaction sets. Many of these variables are not mandatory, and most metabolic models contain only a subset of the variables and identifiers.

Detailed discussion of the different variable categories:

(1) Matrices

Variable	Comment
S	S- matrix, associates metabolites and reactions
RxnGeneMat	matrix for association of reactions and genes

(2) Reaction variables

Variable	Comment
rxns	reaction abbreviations (same as you find in the database)
rxnsNames	full reaction name
subSystems	sorts reactions into ‘metabolic subsystem/pathways’
rxnsKeggID	Kegg ID
rxnECNumbers	E.C. numbers (enzyme identifier)
rxnsConfidenceScore	depends on the support (literature/experimental) that is associated with the reaction (check Nature Protocol Table 2)
rxnsConfidenceEcoIDA	alternative confidence scoring system recon 2 (explained in the supplement of recon 2 paper) Evidence Code Ontology, an ontology created by the Gene Ontology consortium

rxnReferences	references associated with the reaction
rxnNotes	comments on reactions

(3) Concerning metabolites

Variable	Comment
mets	metabolite abbreviations (human metabolism database)
metNames	metabolite Name (human metabolism database)
metFormula	metabolite Formula
metCharge	metabolite charge
metCHEBIID	metabolite identifier
metKeggID	metabolite identifier
metpubChemID	metabolite identifier
metInchiString	metabolite identifier
metHMDB	metabolite identifier
metHepatoNetID	metabolite ID in Hepatonet-applies to those metabolites that originate from Hepatonet
metEHMNID	metabolite ID in EHMNID - applies to those metabolites that originate from Hepatonet

(4) Variables for modeling (reactions)

Variable	Comment
rev	reaction reversibility
rules	gene-proteine-reaction associations
grRules	Gene-Proteine-reaction associations
b	equality constraint (nothing should be consumed or produced, for optimization)
c	vector specifying the objective
lb	lower bound (reaction constraint)
ub	upper bound (reaction constraint)

(5) Boolean vectors (to easily identify reaction sets)

Variable	Comment
ExchRxnBool	redundant for EXRxnBool, does not identify any reaction
EXRxnBool	identifies exchange reactions
DMRxnBool	identifies demand reactions
SinkRxnBool	identifies sink reactions
SIntRxnBool	identifies internal reactions

Double-clicking on the variables in the opened model will open the display of its content, e.g., the vector of lower bounds (model.lb). The S matrix is large and saved as sparse matrix. It can be displayed using the matlab command spy(model.S).

1.2 Infinite constraints

The model reactions can in theory carry a flux that is between zero and ‘infinity’. In the models this ‘infinity’ is replaced by a large numerical value, e.g., ub = 1000 U and lb = -1000 U. The

span of possible fluxes for a reversible reaction can therefore be between an infinite forward flux (1000 U), and an infinite flux in the reverse direction (-1000 U). Exchange reaction stoichiometry is defined such that a negative flux means uptake and a positive flux means secretion.

1.3 Conversion of the theoretical mass

Below an example calculation for the conversion from the theoretical mass ng/ml to mM:

MW(molecular weight) = g/mol

mM = mol*1000/L

LOD = ng/ml

MW(NAD) = 123 g/mol

LOD(NAD)= 3ng/ml

(3ng/ml) / (123 g/mol) | *1e-06

= (0.000003g/l) /(123 g/mol)

= 2.4390e-08 mol/L | *1000

= 2.4390e-05 Mm

1.4 Calculate cell dry weight

The weight of the cell is a necessary input to calculate flux values from the data. If measurements of the dry weight are not available but the wet cell weight, the dry weight can be either assumed to constitute 30% from the wet cell weight [2]. In case the dry weight and the wet weight are not reported in the literature, it can be estimated based on the relative volume difference and comparison with similar cells with reported dry weight in the literature [3].

1.5 Generate metabolic fluxes

The constraints on each reaction (lower bound and upper bound) define how much the model can be maximally consume or release. Flux units depend on the applied constraints. Based on cell concentration, experimental duration and the cellular dry weight fluxes can be calculated Flux = MetConc/(CellConc*CellWeight*T*1000).

1.6 Additional information on scaling of infinite bounds and defined constraints

Metabolite concentrations in cells span multiple magnitudes. The "infinite" bounds need to be higher than the constraints applied to the model, since otherwise these fluxes might limit the model and compromise its predictions.

If any constraints defined in the model, e.g., LOD based qualitative constraints or uptake or secretion fluxes, turn out to be smaller than zero (i.e., $1e^{-8}$, see also COBRA function *getCobraSolverParameters [4]*), the flux unit can be altered to shift the values over the cutoff. This is achieved by multiplying (or dividing) the defined constraints by the same value (e.g., model.lb*10 and model.ub*10). By doing this, also the infinite bounds are increased which might be unnecessary. Additionally, the coefficients of metabolites in the biomass objective

function need to be multiplied or divided by the same factor to retain the relationship (growth rate = ln(2)/td) of growth rate to doubling time (dt). This can be done by multiplying (or dividing) the column of the S matrix that corresponds to the biomass reaction by the same value as was used to scale the remaining constraints.

However, scaling might cause other constraints to exceed the constraints. This can be solved by increasing the infinite bounds. However, increasing the infinite bounds increases the solution space. An increase of the infinite bounds only does not change the unit fluxes are reported in.

1.7 Additional remarks on the integration of Gene Expression data

Although generation a functional model using an algorithm that assumes gene regulation in order to fulfil the requirement of a functional model is quite fast, this automation comes with drawbacks. Thus, it might be worthwhile to invest the time to manually curate the network or explore different statistical thresholds to generate a more conservative reaction set [3, 5]. The trade-off is less reduction of the internal network redundancy for less network gaps. A less stringent threshold could, in combination with the constraints from the metabolomic data, describe a good compromise. However, the best strategy might be different between data sets. At last, manual curation is always also an opportunity to gain insight into the data, rather than just a necessary, time consuming task. In fact, knowledge about the data can help later on during interpretation of the simulation results, e.g., why one pathway might be chosen over another and might help to uncover "method-driven" false predictions.

1.8 Additional remarks on "Sampling the solution space"

During sampling analysis every *i*th point (i.e., each one flux vector) are collected while performing a random-walk with "random" direction and step length, through the solution space (Figure 8). *i* describes the number of points skipped between two collected points, and defines the mixing of the points. Files of points are saved after a defined number of points have been collected. In case more points need to be collected, use the last point of the last file saved to generate more points. Before starting the sampling, increased the java heap space in Matlab to the maximum. The duration of the analysis depends on the size of the model, the computer, the number of collected points, and number of skipped points.

When do you know that you have sampled enough: In order do know when one has sampled "enough", it is good to look at the histograms (Figure 8), which can be generated using the function *summarizeSamplingResults* from the model and the sampling results. A good evaluation is to look at the probability distribution and specifically at the shape of the distribution. If the distributions are evenly round-shaped and no edges stick out this is an indication that enough sampling points have been collected (which of course also varies with the binning of the histogram, Figure 8B). A good test is to compare the distributions e.g. for 60x 5000 points and 65x5000, … , and so on. If the distributions keep improving or the shape keeps changing, this is a sign that one should continue sampling (Figure 8B, D). For a previous analysis 100 x 5000 points were generated [3].

It should be noted that one can only obtain regular distributions for bounded reactions (Figure 8B, C) and that the distributions will continue to change for unbounded (or loop) reactions (see Box 1). One way to evaluate the sampling is to check if the distributions converge with the minFlux and maxFlux values obtained through flux variability analysis.

Supplementary Table 1: Appeal from a computational biologist to the metabolomics community. The table lists points that simplify the integration of metabolomics data sets into the model context.

**Consideration**	**Explanation**	**Solution**
Report of standard metabolite identifiers	Simplifies matching the namespace of the metabolic model and the data and prevents errors in the translation.	e.g., HDMI.<br>Refer to [1]
Multiple time points	Constraint-based modeling uses metabolic fluxes, i.e., the change of metabolite concentration over time.	Refer to [6]
High coverage	The better the metabolic model can be defined, the more accurate will be the predictions. The human model includes currently ~800 metabolites that can be exchanged with the extracellular environment. Missing quantification can be compensated by a well-defined culture medium.	Refer to [6]
Quantification of metabolites	High coverage in combination with quantification provides an ideal prerequisite for intra-model analysis.	Refer to [7]

2 References

1. Thiele, I., et al., *A community-driven global reconstruction of human metabolism.* Nat Biotechnol, 2013. **31**(5): p. 419-25.
2. Guyton A.C., H.J.E., *Text book of medical physiology*. 2000: W.B. Saunders company.
3. Aurich, M., et al., *Prediction of intracellular metabolic states from extracellular metabolomic data.* Metabolomics, 2015. **11**(3): p. 603-619.
4. Schellenberger, J., et al., *Quantitative prediction of cellular metabolism with constraint-based models: the COBRA Toolbox v2.0.* Nat Protoc, 2011. **6**(9): p. 1290-307.
5. Aurich, M.K. and I. Thiele, *Contextualization procedure and modeling of monocyte specific TLR signaling.* PLoS One, 2012. **7**(12): p. e49978.
6. Paglia, G., et al., *Monitoring metabolites consumption and secretion in cultured cells using ultra-performance liquid chromatography quadrupole-time of flight mass spectrometry (UPLC-Q-ToF-MS).* Anal Bioanal Chem, 2012. **402**(3): p. 1183-98.
7. Jain, M., et al., *Metabolite profiling identifies a key role for glycine in rapid cancer cell proliferation.* Science, 2012. **336**(6084): p. 1040-4.
