# Supplementary material for: MetaboTools: A Comprehensive Toolbox for Analysis of Genome-Scale Metabolic Models
Source: Front Physiol. 2016 Aug 3;7:327. doi: 10.3389/fphys.2016.00327 (PMC4971542; doi:10.3389/fphys.2016.00327)
Supplement: Supplementary file 2 [file DataSheet2.PDF]

MetaboTools: A comprehensive toolbox for analysis of genome-scale metabolic models.

Maike K. Aurich[1] Ronan M.T. Fleming[1], and Ines Thiele[1*]

1 Luxembourg Centre for Systems Biomedicine, University of Luxembourg, Esch-sur-Alzette, Luxembourg.

∗ Corresponding author: Ines Thiele, Luxembourg Centre for Systems Biomedicine, University of Luxembourg, 7, avenue des Hauts-Fourneaux, L-4362 Esch-sur-Alzette, E-mail: ines.thiele@uni.lu

Tutorial I: Workflow for the integration of semi-quantitative extracellular metabolomic data into the network context.

This tutorial describes step-by-step the generation of two condition-specific, lymphoblastic leukemia cell line models based on semi-quantitative metabolomic data as in [1]. All necessary code and input data is provided with this tutorial (runTutorial_I.m). The boxes illustrate the formulation of the functions including inputs and outputs that need to be defined. After reading a section in the tutorial, execute the respective section in runTutorial_I.m and check the format of inputs and outputs at each step. Some of the functions require saving and retrieving files (i.e., clone1.log, and A.mat). Make sure that you have writing rights in the working directory when executing the files, or move to a folder where you hold the rights. In Matlab, text is entered as follows: variable_name = 'first_entry'; 'second_entry'; ....; Numbers are entered as follows: variable_name =[0;1;0;...]; More information about the inputs and outputs of the functions can be obtained by typing 'help FUNCTIONNAME' into the command window in Matlab.

Requirements of software:

- Matlab (Mathworks, Inc)
- COBRA toolbox (https://github.com/opencobra/cobratoolbox, [2])
- A linear programming solver

Preview on consecutive steps

(A) **Apply constraints to the model**

- *setMediumConstraints*: Shaping the model's environment according to the medium concentrations.
- *calculateLODs*: Transform units of instrumental limits of detection (LODs).
- *defineUptakeSecretionProfiles*: Identify sample specific sets of consumed (uptake) and released (secretion) metabolites.
- *calculateQuantitativeDiffs*: Define (semi-)quantitative differences of commonly consumed and released metabolites based on the relative differences in the data.
- *setQualitativeConstraints*: Enforce sample-specific metabolite uptake and secretion profile.
- *setSemiQuantConstraints*: Apply the (semi-)quantitative constraints.
- *setConstraintsOnBiomassReaction*: Apply constraints on biomass production.
- *integrateGeneExpressionData*: Apply constraints based on sets of unexpressed genes.

(B) **Generate condition specific models**

- *extractConditionSpecificModel*: Generate contextualized models.

(C) **Analyze the condition specific models**

- *performSampling*: Run a sampling analysis.
- *summarizeSamplingResults*: Illustrate the results of the sampling analysis.

Start of the tutorial

In this tutorial, contextualized models of two lymphoblastic leukemia cell lines, CCRF-CEM and Molt-4 cells, will be generated by integrating semi-quantitative metabolomic data, transcriptomic data, and growth rates. After the generation of two contextualized submodels, the solution space of these models will be analyzed by sampling analysis [1]. Please note that in order to keep the computation time of this tutorial short, fewer sampling points will be collected and results can divert from those reported in [1].
This tutorial only covers the computational steps of the workflow. The name space of model and data were already mapped and transport and exchange reactions were added to the generic model deemed *starting_model*. All variables necessary to execute the MetaboTools functions are specified in the run_tutorial_I.m file. All that needs to be specified is the path to the location where the cobratoolbox is saved (pathToCOBRA), where the results of the analysis will be saved (path), and the solver (solver) that is installed on your computer. The boxes in each section are for illustration of the functions only, the respective inputs have been filled in, in the run_tutorial_I.m file. Make sure the MetaboTools are in the Matlab path.
Specify solver, path, and pathToCOBRA (see Box 1 + run_tutorial_I.m) and execute the sections on 'set and check solver' and 'load and check input is loaded correctly', and 'check output path and writing permission' to make sure path and solver are set correctly.

1 Shaping the model's environment

Cells have a limited supply of nutrients under most experimental conditions. When relying on semi-quantitative metabolomics data, the exact change of concentrations, which is metabolite uptake or secretion, is unknown. In this case only the relative difference in uptake or secretion is known. In order to integrate the data, a baseline needs to be established, that has a biological reference as compared to the infinite bounds of the model. Based on this "baseline", the relative differences can be that distinguish two samples can be incorporated into the model.
In our example, the baseline is established by "placing" the model in an environment that supplies the same amount of nutrients to the model as were maxiamally available to one cell in the experiment. The maximal uptake of a metabolites is limited or constrained according to the concentration of metablites in the cell culture medium, that could have been available to one cell per time unit using the function *setMediumConstraints* (see Box 1 + execute code from run_tutorial_I.m). To run the function *setMediumConstraints*, a number of input variables need to be defined.
The function *setMediumConstraints* can be used to alter the infinite constraints. In our example [1], the cells were grown in RPMI medium with an addition of 2 mM of Glutamax, a stabilized form of glutamine (Variables: *medium_composition* and *met_Conc_mM*). The metabolite concentrations are converted into flux values, using the specific cell concentration (*cellConc* = 2.17*1e6), g dry weight of one cell (*cellWeight* = 3.645e-12), the duration of the experiment in hours (*t* = 48).
Because the size of the applied constraints was rather small in this example, we reduced the size of the infinite constraints in the starting model from *current_inf* = 1000 to *set_inf* =500.

Box 1

Input for matlab:

```
>> initCobraToolbox

>> solver = '...ADD YOUR SOLVER...'
>> pathToCOBRA = '...ADD YOUR PATH TO cobratoolbox...';
>> path = '...ADD YOUR PATH TO OUTPUT FOLDER...';
```

set and check solver
>> changeCobraSolver(solver,'LP');

Load and check input
>> load('starting_model.mat');

>> [modelMedium,basisMedium] = setMediumConstraints(starting_model, set_inf, current_inf, medium_composition, met_Conc_mM, cellConc, t, cellWeight, mediumCompounds, mediumCompounds_lb, customizedConstraints, customizedConstraints_ub, customizedConstraints_lb, close_exchanges);
>> clearvars -EXCEPT modelMedium basisMedium

Constraints were added to limit the uptake by the model of a number of compounds that were not defined in the medium (*mediumCompounds*). Uptake of these metabolites was restricted uniformly to *mediumCompounds_lb* =-100. Upper and lower bounds can also be individually defined for exchange reactions specified in *customizedConstraints* (*customizedConstraints_ub* and *customizedConstraints_lb*), e.g., to define the maximum oxygen uptake and prevent secretion of oxygen [1].
The last line in the box cleans up your workspace except for those variables needed at a later stage in this tutorial.
The result of step 1 is a model (*modelMedium*) that is restricted to defined influx of metabolites and based on the culture medium, *mediumCompounds*, and *customizedConstraints*.
One way to check that this step had an impact on the model is to compare the growth rate that can be maximally achieved by the *modelMedium* as compared to the *starting_model* (use the COBRA toolbox function *optimizeCbModel*). The growth rate should be reduced, since the carbon sources that the model can use to grow have been limited (i.e., 0.9228 mmol*gdrw^{-1}*hr^{-1} as compared to 100 mmol*gdrw^{-1}*hr^{-1}).

2 Transform units on instrumental limits of detection (LODs)

We used the detection limits to define the lower limits of uptake and secretion in the leukemia cell line models [1]. To prepare the use of the values, the LODs *lod_ngmL* were transformed from ng/ml to mM using the theoretical mass *theo_mass* and the MetaboTools function *calculateLODs*. Later, the mM concentrations will be converted into flux values, which will be applied to the bounds to enforce metabolite uptake and secretion.

Box 2
Input for matlab:
>>[lod_mM] = calculateLODs(theo_mass,lod_ngmL);

---

3 Define uptake and secretion profiles

Generate the individual uptake and secretion profiles for the preliminary CCRF-CEM and the Molt-4 model. The original data is specified by *input_A*, *input_B*, and *data_RXNS*. To run the function

defineUptakeSecretionProfiles, empty fields in the *input_A* and *input_B* were filled (value = 3000). The tolerance to distinguish a real change (samples to control) and will be set to 5 % (*tol* = 0.05). The function allows one to add and exclude exchange reactions from the generated uptake and secretion profiles (*add_secr*, *add_upt*,*exclude_upt* and *exclude_secr*). In the case of the leukemia cell lines, we excluded metabolite exchanges such as glutamine and the alanine since the direction of exchange was not certain [1]. Thus, the options of the function allow the alternation of the profiles if needed.

Box 3

Input for matlab:

```
>> [cond1_uptake, cond2_uptake, cond1_secretion, cond2_secretion, slope_Ratio] = defineUptakeSecretionProfiles (input_A, input_B, data_RXNS, tol, essAA_excl, exclude_upt, exclude_secr,add_secr, add_upt);
```

The profiles produced by *defineUptakeSecretionProfiles* were further altered based on additional criteria, e.g., directionality of change in controls and samples was opposite and from that resulted no change, or small signal differences between changes not picked up by the tolerance we decided to reject (*remove_secretion*,*added_secretion*,*remove_uptake*,*add_uptake*, [1]). The code in Box 4 shows how the alternations were performed.

Box 4

Input for matlab:

```
>> cond2_secretion = [cond2_secretion; add_secretion];
>> cond2_secretion(find(ismember(cond2_secretion, remove_secretion)))=[];
>> cond2_uptake = [cond2_uptake; add_uptake];
>> cond2_uptake(find(ismember(cond2_uptake, remove_uptake)))=[];
```

As a result of this step, the uptake and secretion profiles have been corrected.

4 Calculate Semi-quantitative Differences

The relative differences in metabolite uptake and secretion can automatically be defined using the function *calculateQuantitativeDiffs* based on the outputs of the previous steps. The input variable *ex_RXNS* specifies metabolite exchange reactions in the same order as the LODs were specified.

Box 5

Input for matlab:

```
>> [cond1_upt_higher, cond2_upt_higher, cond2_secr_higher, cond1_secr_higher,
cond1_uptake_LODs, cond2_uptake_LODs, cond1_secretion_LODs, cond2_secretion_LODs] = calculateQuantitativeDiffs(data_RXNS, slope_Ratio, ex_RXNS, lod_mM, cond1_uptake, cond2_uptake, cond1_secretion, cond2_secretion);
```

Again you might want to remove exchange reactions (e.g., the metabolite 'anth' was not detected in the 48 hr samples, Figure 1). It could be assumed that all of it (below the LOD) was consumed, however that

would have meant a 1975% relative difference between Molt-4 and CCRF-CEM cells. Thus, the metabolite was removed (*remove*) from the set of metabolite exchange reactions that were to be constrained based on the relative difference.

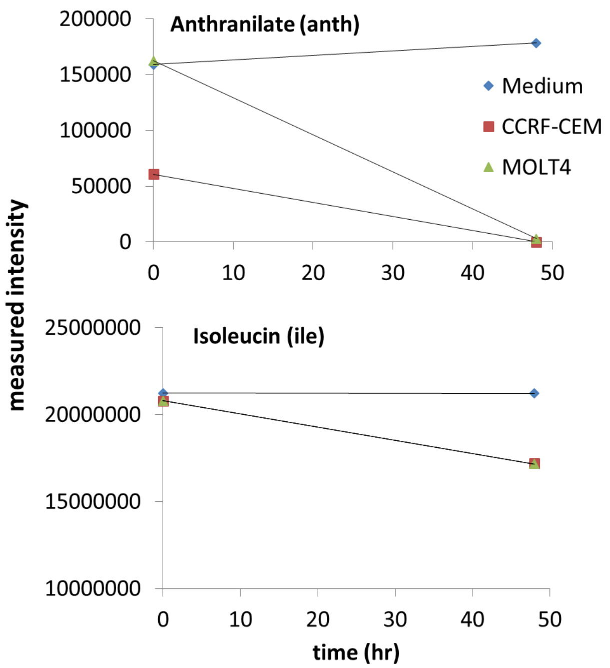

Figure 1. Removed quantitative differences

Box 6

Input for matlab:

```
>>A = [];
>>for i=1:length(cond2_upt_higher)
>> if find(ismember(remove, cond2_upt_higheri,1))>0;
>> A = [A;i];
>> end
>>end
>>cond2_upt_higher(A,:)=[];
```

5 Define Qualitative Constraints

Enforce the direction of exchange to be uptake or secretion by setting the lower bound of the exchange reactions to the flux calculated from the individual detection limit of the metabolite using the function *setQualitativeConstraints*. The basic medium composition *basic_Medium* was already defined by *setMediumConstraints*. Using the detection limits has the advantage that the minimal flux value is varied according to the individual chemico-physical properties of the metabolite.

If there are other metabolites that should not be closed and are not yet considered in the medium composition, they can be defined individually (*ambiguous_metabolites*). We added metabolites for which it was not clear if and in what quantity they were exchanged by the cell to *ambiguous_metabolites*. This step is performed individually for each model.

Box 7

Input for matlab:

```
>> cellConc = 2.17*1e6;
>> t= 48;
>> cellWeight = 3.645e-12;
>> [model_A] = setQualitativeConstraints(modelMedium, cond1_uptake,
cond1_uptake_LODs, cond1_secretion, cond1_secretion_LODs, cellConc, t, cellWeight, ambiguous_metabolites,
basisMedium);
>> [model_B] = setQualitativeConstraints(modelMedium, cond2_uptake,
cond2_uptake_LODs, cond2_secretion, cond2_secretion_LODs, cellConc, t, cellWeight, ambiguous_metabolites,
basisMedium);
```

As a result of the step, you should have two models (*model_A* and *model_B*) of the size of the starting model, each constrained with the cell line specific uptake and secretion profile (qualitative).

6 Apply semi-quantitative constraints

After enforcing directions of metabolite exchange based on the input data, quantitative differences between commonly consumed and released metabolites will be established using the function *setSemiQuantConstraints*. The function relies on outputs from previous steps. This function only needs to be executed once.

Box 8

Input for matlab:

```
>> [modelA_QUANT,modelB_QUANT] = setSemiQuantConstraints(model_A,model_B, cond1_upt_higher,
cond2_upt_higher, cond2_secr_higher,cond1_secr_higher);
```

As a result, the preliminary models have different requirements for metabolites consumed and released by both cell lines.

7 Apply growth constraints to the models

Apply doubling times (*dT*) of the respective cell lines as constraints to further distinguish the metabolic models, while accounting for a 20% error (*tolerance* = 20) to establish upper and lower bound of the biomass objective function (*of*). This step has to be done separately for the two models.

Box 9

Input for matlab:

```
>> of = 'biomass_reaction2';
>> tolerance = 20;
% Make Molt-4 model
>> dT= 19.6;
>> [model_A_BM] = setConstraintsOnBiomassReaction(modelA_QUANT, of, dT, tolerance);
```

```
% Make CCRF-CEM model
>> dT= 22;
>> [model_B_BM] = setConstraintsOnBiomassReaction(modelB_QUANT, of, dT, tolerance);
```

As a result, the models are now constrained down to the specific growth rate observed for each cell line [1].

8 Integrate transcriptomic data

Transcriptomic data can be integrated to further contextualize the models. The function *integrateGeneExpressionData* uses a set of absent genes (*data_genes*), and constrains the reactions associated with this gene to zero.

Box 10

Input for matlab:

```
>> [model_A_GE] = integrateGeneExpressionData(model_A_BM, dataGenes);
>> [model_B_GE] = integrateGeneExpressionData(model_B_BM, dataGenes);
```

With the completion of this step, all constraints have been applied as in [1].

9 Generate condition-specific models

During the previous steps, many exchange reactions were disabled, leading to the blockage of flux through associated pathways. In this step, a submodel will be extracted, which comprises of the reactions and pathways that still carry flux, using the function *extractConditionSpecificModel*. The active reaction set is hereby defined by running flux variability analysis. A threshold defines the cutoff for calling a flux zero (threshold = 10e-6).

Box 11

Input for matlab:

```
>> [model_Molt] = extractConditionSpecificModel(model_A_GE, threshold);
>> [model_CEM] = extractConditionSpecificModel(model_B_GE, threshold);
>> clearvars -EXCEPT model_Molt model_CEM modelMedium
```

This step has completed the generation of the contextualized models of Molt-4 and CCRF-CEM cells.

10 Plot network connectivity

To get an impression of the differences between the models the connectivity can be compared among each other as well as to the generic model (*modelMedium*) using the function *networkTopology*.

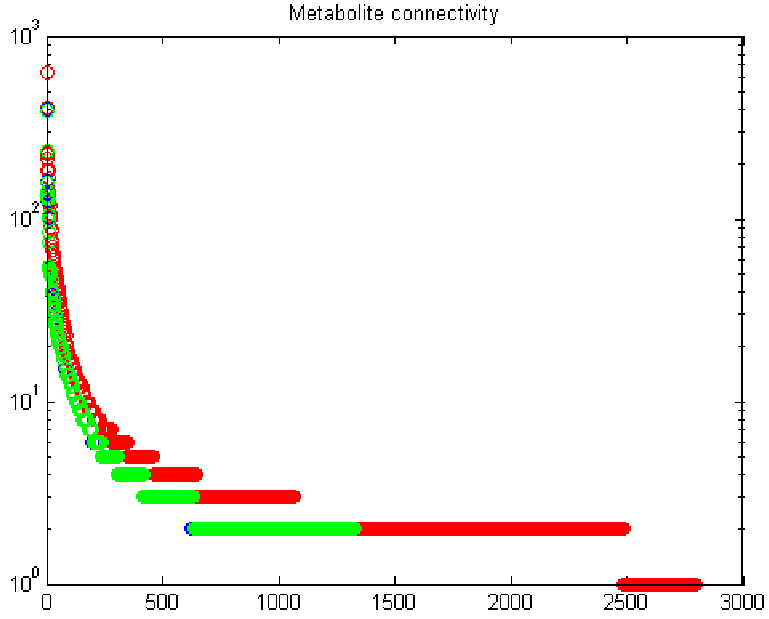

Figure 2. Comparison of Metabolite connectivity.

Box 12

Input for matlab:

```
>> [MetConn,RxnLength] = networkTopology(modelMedium);
>> [MetConnA,RxnLengthA] = networkTopology(model_Molt);
>> [MetConnB,RxnLengthB] = networkTopology(model_CEM);
>> MetConnCompare = sort(MetConn,'descend');
>> MetConnCompareA = sort(MetConnA,'descend');
>> MetConnCompareB = sort(MetConnB,'descend');


% Plot metabolite connectivity
>> figure
>> semilogy(sort(MetConnCompare,'descend'),'ro')
>> hold
>> semilogy(sort(MetConnCompareA,'descend'),'bo')
>> semilogy(sort(MetConnCompareB,'descend'),'go')
>> title ('Metabolite connectivity')
```

The illustration shows a clear distinction in the metabolite connectivity of the contextualized metabolic models of CCRF-CEM and molt-4 cells as compared to the generic model (Figure 2). The difference between the two contextualized models on the other hand, is rather small. This similarity attributes to the general similarity of the metabolomic and transcriptomic data. The metabolites in the exchange profiles of the two cell lines were largely overlapping, as were the sets of absent genes.

11 Perform sampling analysis

We will continue to look at the functional differences predicted for the two contextualized cell line models. Perform a sampling analysis for the two condition-specific models by using the function *performSampling*. In the initial phase warmup points are generated (*warmupn* = 2000). Subsequently, the actual sampling is performed, gathering 1000 points in each file (*nFiles* = 10, *pointsPerFile* = 1000). To support the mixing of the collected points, 500 steps are skipped between two collected points (*stepsPerPoint* = 500). The file numbering will start at 1 (*fileBaseNo* = 0), and the sampling time should not exceed a specified time window (*maxTime* = 3600000). Each output file is saved under the specified name *fileName* = 'sampling_modelX', at the specified location *path* = '*Copy in path to your output directory*'. Perform the sampling separately for the two models.

Box 13

Input for matlab:

```
>> warmupn = 2000;
>> pointsPerFile = 1000;
>> stepsPerPoint = 500;
>> fileBaseNo = 0;
>> nFiles = 10;
>> maxTime = 3600000;
>> path = 'Copy in path to your output directory';
>> fileNameA = 'sampling_modelA';
>> fileNameB = 'sampling_modelB';
>> performSampling(model_Molt, warmupn, fileNameA, nFiles, pointsPerFile, stepsPerPoint, fileBaseNo,
maxTime, path);
>> performSampling(model_CEM, warmupn, fileNameB, nFiles, pointsPerFile, stepsPerPoint, fileBaseNo,
maxTime,path);
```

Subsequently, the function *summarizeSamplingResults* can be applied to illustrate the results of the sampling analysis. The function takes as input the contextualized models (*model_Molt*, *model_CEM*), and loads the files generated in the previous steps. We will illustrate four reactions that were important to the main observation (*show_rxns*). Some parameters of the illustration can be customized, i.e., fond size (*fonts*=8), the number of histograms per page (*hist_per_page*=4), and binning of the probability distribution (*bin*=30). The association of the illustrated reactions with genes of interest (*dataGenes*) can be automatically indicated in the histogram, e.g., essential genes or differentially expressed genes.

Box 14

Input for matlab:

```
>> [stats, statsR] = summarizeSamplingResults(model_Molt, model_CEM, path, nFiles, pointsPerFile,
starting_model, dataGenes, show_rxns, fonts, hist_per_page, bin, fileNameA, fileNameB);
```

The resulting histograms illustrate the main results observed in [1]. The shapes of the histograms are not quite even (Figure 3). This illustrates that not enough sampling points have been collected. Nevertheless, the distributions and medians of the two cell lines reveal a shift indicating a difference in the use of glycolysis and the electron transport chain by the two contextualized models.

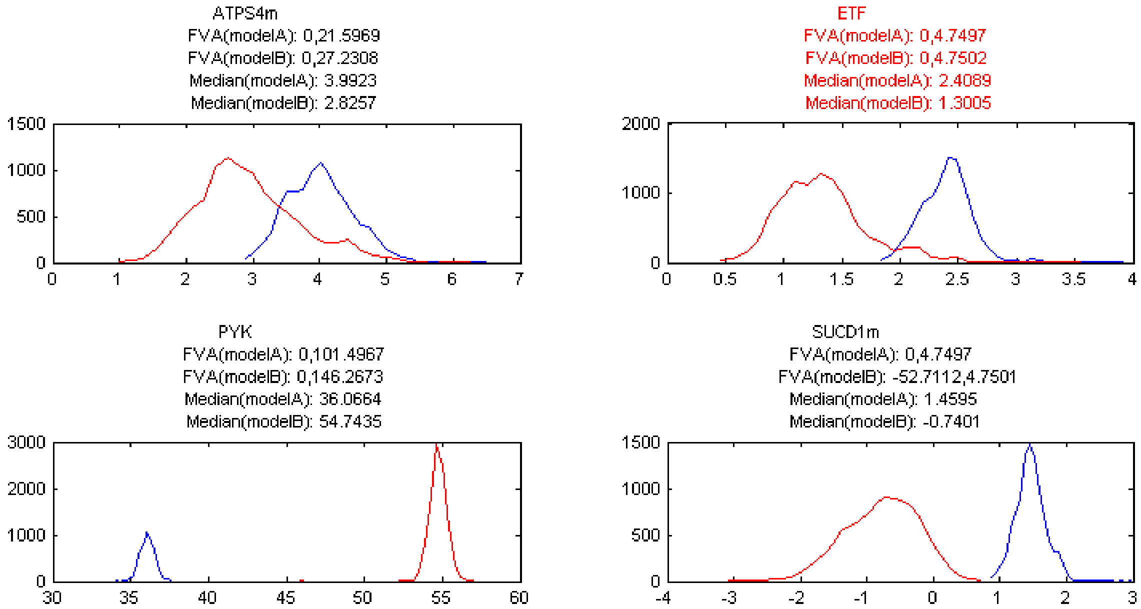

Figure 3. Histograms of sampling analysis.

References

1. Aurich MK, Paglia G, Rolfsson Ó, Hrafnsdóttir S, Magnúsdóttir M, et al. (2015) Prediction of intracellular metabolic states from extracellular metabolomic data. Metabolomics 11: 603–619.

2. Schellenberger J, Park J, Conrad T, Palsson B (2010) BiGG: a Biochemical Genetic and Genomic knowledgebase of large scale metabolic reconstructions. BMC Bioinformatics 11: 213.
