# Supplementary material for: MetaboTools: A Comprehensive Toolbox for Analysis of Genome-Scale Metabolic Models
Source: Front Physiol. 2016 Aug 3;7:327. doi: 10.3389/fphys.2016.00327 (PMC4971542; doi:10.3389/fphys.2016.00327)
Supplement: Supplementary file 3 [file DataSheet3.PDF]

MetaboTools: A comprehensive toolbox for analysis of genome-scale metabolic models.

Maike K. Aurich[1] Ronan M.T. Fleming[1], and Ines Thiele[1*]

1 Luxembourg Centre for Systems Biomedicine, University of Luxembourg, Esch-sur-Alzette, Luxembourg.

∗ Corresponding author: Ines Thiele, Luxembourg Centre for Systems Biomedicine, University of Luxembourg, 7, avenue des Hauts-Fourneaux, L-4362 Esch-sur-Alzette, E-mail: ines.thiele@uni.lu

Tutorial II: Workflow for the integration of quantitative extracellular metabolomic data into the network context.

This tutorial describes in a step-by-step manner the generation of condition-specific cancer cell line models based on quantitative metabolomic data [1]. All necessary code and input data is provided with this tutorial (runTutorial_II.m). Boxes illustrate the formulation of the functions including inputs that need to be defined and outputs that are generated by the function. After reading a section in the tutorial, execute the respective section in runTutorial_II.m and carefully inspect the format of inputs and outputs at each step. Some of the functions require saving and removing files (i.e., clone1.log, and A.mat). Make sure to have writing rights in the working directory when executing the code or move to a folder where you hold the rights.

Requirements of software:

- Matlab (Mathworks, Inc)
- COBRA toolbox (https://github.com/opencobra/cobratoolbox, [2])
- A linear programming solver. ILOG cplex (solver = 'cplex_direct') is required to run *generateCompactExchModel*, which is also called by

setQuantConstraints. The other steps can be executed by using tomlab cplex or gurobi.

Preview on consecutive steps

(A) **Prepare and apply constraints to the starting model**

- *setMediumConstraints*: Apply basic constraints on the starting model
- *prepIntegrationQuant*: Prepare the uptake and secretion profiles based on an input matrix of fluxes.
- *checkExchangeProfiles*: Summarize exchange profiles across samples.

(B) **Generate condition-specific models**

- *setQuantConstraints*: Integrate quantitative metabolomic data and use minExCard to predict condition-specific models that contain only a minimal set of exchange reactions in addition to those defined through the data.

(C) **Analyze the condition-specific models**

- *statisticsAddedExchanges* and *mkTableOfAddedExchanges*: Summarize the minimal set of metabolite exchange reactions predicted by minExCard across the set of models.
- *analyzeSingleGeneDeletion*: Perform and summarize the results of the single gene deletion.
- *checkEffectRxnKO*: Check which reactions that are associated with an essential gene need to carry flux.
- *predictFluxSplits*: Compare strategies of metabolite production across a model set. Compare ATP yields across a model set.

- *performPPP* and *illustrate_ppp*: Compare and illustrate predicted response to changes in metabolite uptake or secretion.
- *makeSummaryModels*: Generate union and intersect model from a set of models.

Start of the tutorial

In this tutorial, six condition-specific models of cancer cell lines will be generated and analyzed. Again, the manual association of metabolites between the data and the model was already performed and transport and exchange reactions have been added.
In a first step, the cobra toolbox needs to be initiated and the solver needs to be set. For this tutorial, a linear programming (LP) solver and a quadratic programming (QP) solver are required. The model building requires ILOG CPLEX (solverQuant = 'cplex_direct'). A different solver can be used for the remaining steps.
Inputs, outputs, and the code to execute the functions are specified in runTutorial_II.m. Execute the code for individual sections after reading the respective part in this tutorial (e.g., mark the part that should be executed in the runTutorial_II.m and press F9 or copy and paste the code into the command window). The variables will appear in the workspace. Inspect the content and format of the inputs and outputs. By double-clicking on the individual variables (in the matlab workspace), they will be opened in matlab (Figure S1).
Additional information on the inputs and the outputs of a function can be displayed by typing 'help ...' followed by the function name into the Matlab command window (Box 1).
The unit of fluxes is fmol/cell/hr. Fluxes that are smaller than 1e-6 will be considered as zero throughout the tutorial (Box 1).

Box 1

Input for matlab:

```
>> clear
>> initCobraToolbox
```

Default parameters:

```
>> tol = 1e-6;
```

Get more information on a *function*. Define your solver according to the inputs listed in *changeCobraSolver*:

```
>> help changeCobraSolver
```

Define and set your LP and QP solver:

```
>> solver ='...specify your solver';
```

Define the path to the cobratoolbox.

```
>> pathToCOBRA = '.. ADD YOUR PATH TO cobratoolbox';
```

Define location to save outputs:

```
>> path ='...your path';
```

Define and check the solver

```
>> changeCobraSolver(solver,'LP');
```

Load and check tutorial input

```
>> load([pathToCOBRA '/starting_model']);
```

After defining the solver, pathToCOBRA, and path (can be same as pathToCOBRA), execute the sections on 'define and check the solver' and 'load and check tutorial input is loaded correctly' in runTutorial_II.m. After the model is loaded and the LP and the QP solver are set, proceed with the rest of the tutorial.

1 Shaping the model's environment

Use the function *setMediumConstraints* to add a set of basic constraints (*mediumCompounds*) to the model, e.g., uptake of ions (*lb* =-100 U). Increase the infinite constraints from 1000 U to 2000 U to leave enough space between the integrated constraints and the infinite bounds (*current_inf* = 1000, *set_inf* = 2000). In comparison to the first tutorial, the uptake of metabolites will be directly constrained based on the data and a medium composition is not considered (*medium_composition* = {}), and do not need to be converted into fluxes (*met_Conc_mM*, *cellConc*, and *cellWeight*). Furthermore, undefined exchange reactions will not closed in this step (*close_exchanges* = 0). After executing the function and inspecting the inputs and outputs clearvars -EXCEPT can be used to clean up the workspace from variables that are no longer needed.

Box 2

Input for matlab:

```
>> [modelMedium,basisMedium] = setMediumConstraints(model, set_inf, current_inf, medium_composition, met_Conc_mM, cellConc, t, cellWeight, mediumCompounds, mediumCompounds_lb, customizedConstraints, customizedConstraints_ub, customizedConstraints_lb, close_exchanges);
>> clearvars -EXCEPT modelMedium model
```

The output of *setMediumConstraints* will be *modelMedium*. Check out the newly applied bounds, double-click on the *modelMedium* in the workspace, and display the lower bounds by double-clicking on 'model.lb' (Figure S1). Compare the bounds to those of the generic model (*model*), e.g., by pasting them into excel along with the reaction names.

2 Define uptake and secretion profiles

Use the function *prepIntegrationQuant* to define the individual uptake and secretion profile for each of the four samples based on the data (tuto-

rial_II_data.mat) and a model (starting_model.mat). The individual uptake and secretion profiles will be saved under the sample name (*samples*) to the specified location (*path*). Exchanges that cannot be consumed (*test_max*, *test_min*), will be sorted out and will not be part of the generated exchange profiles. The output file will contain, next to the names of uptake exchanges and secretion exchanges, the flux values (U) that will be integrated into the model in the next step. The flux values (U) for the upper bounds and the lower bounds will be the measured flux plus and minus a user-defined error range (*variation* = 20).

Box 3

Input for matlab:

```
>> load([path 'tutorial_II_data']);
>> prepIntegrationQuant(model, metData, exchanges, samples, test_max,
test_min, path, tol, variation);
>> load([path 'PC_3.mat']);
```

Load one of the output files from the path location into the workspace, e.g., PC_3.mat. The variable *secretion* contains all secreted metabolites of the sample. The variable *secr_value* lists the original flux values, the values for the new upper bound and the value for the new lower bounds. Similarly, the variable *uptake* lists all consumed metabolites, and *uptake_value* lists original and new reaction constraints for all metabolites consumed by the cells. In case of PC-3, 42 uptakes 58 secretions will be integrated into the model (Table 1).

2.1 Prepare table to check exchange profiles

The function *checkExchangeProfiles* loads the individual exchange profiles generated in the previous step, from the specified location (*path*) and provides an overview of the exchanges in each sample (Box 4, Table 1). The number *nmets* specifies the size of the output table (maximum *nmets* = length(exchanges)). A summary table lists the minimal and the maximal number of metabolites consumed and released (*minMax*). Additionally,

summaries of the uptake profiles and secretion profiles will be generated (*mapped_secretion*, *mapped_uptake*).

Box 4

Input for matlab:

>> [mapped_exchanges, minMax, mapped_uptake, mapped_secretion] = checkExchangeProfiles(samples, path ,nmets);

Table 1. Overview of the exchange profiles (*mapped_exchanges*) generated by *checkExchangeProfiles* across *samples*

	PC-3	PC-3-2	IGROV1	IGROV1-2	SNB-19	SNB-19-2
**secretion**	58	58	53	57	46	43
**uptake**	42	42	47	43	51	52
**sum**	100	100	100	100	97	95

Based on the outputs of *checkExchangeProfiles*, the exchange profiles can be easily inspected for errors.

3 Generate cancer cell models

Use the function *setQuantConstraints* to integrate the uptake and secretion profiles and generate condition-specific metabolic models for each sample. A lower bound can be applied (*minGrowth*) to the biomass reaction (*obj*). The function allows the definition of metabolites that should not be consumed (*no_uptake*) or not secreted (*no_secretion*), and thus allows adding constraints iteratively if minExCard predicts the addition of metabolite exchange reactions which are unlikely from a biological point of view. The variable *medium* defines exchange reactions that should be excluded from minimization of exchanges (in addition to those of defined in the individual uptake and secretion, and thus will be retained in the output model). Here, no exchanges will be excluded. The variable *addExtraExch* allows the definition of metabolite exchanges and flux values that are added to the upper and lower bounds. The output of *setQuantConstraints* is automatically saved to a specified location (*path*). Note that the solver ='cplex_direct' is required for this step.

Box 5

Input for matlab:
>> solver = 'cplex_direct';
>> changeCobraSolver(solver,'LP');
>> [ResultsAllCellLines,OverViewResults] = setQuantConstraints(model, samples, tol, minGrowth, obj, no_secretion, no_uptake, medium, solver, addExtraExch, addExtraExch value, path);
>> solver = 'your solver';
>> changeCobraSolver(solver,'LP');

Table 2. Overview of the condition-specific cell line models

cell line	added reactions	reactions	metabolites	genes	maximal growth rate	exchange reactions	O2 required
PC-3	16	2176	1463	1526	13.5607	116	1
PC-3-2	16	2174	1461	1526	13.683	116	1
IGROV1	17	2179	1464	1525	15.4836	117	1
IGROV1-2	18	2180	1464	1525	15.3528	118	1
SNB-19	22	2169	1456	1526	0.62777	119	1
SNB-19-2	21	2169	1459	1530	0.48036	116	1

4 Analyze added exchanges

Use the function *statisticsAddedExchanges* to get a table summarizing the exchange reactions that were added to the models (*Ex_added_all_unique*, Box 6 and Table 3). The function*mkTableOfAddedExchanges* generates a table that summarizes the reactions added to individual models (*Added_all*).

Box 6 ___

Input for matlab:
>> [Ex_added_all_unique] = statisticsAddedExchanges(ResultsAllCellLines, samples);
>> [Added_all] = mkTableOfAddedExchanges(ResultsAllCellLines, samples, Ex_added_all_unique);

Table 3. Summary of the added exchanges, and directions of exchange in the models (*Ex_added_all_unique*)

**Exchange reaction**	**models**	**secretion**	**uptake**
EX-2hb(e)	6	secretion	[]
EX-bilglcur(e)	6	secretion	uptake
EX-o2(e)	6	[]	uptake
EX-tdchola(e)	6	secretion	[]
EX-thmmp(e)	6	[]	uptake
EX-triodthysuf(e)	6	secretion	[]
EX-urea(e)	6	secretion	[]
EX-dmhptcrn(e)	5	secretion	[]
EX-phyt(e)	5	[]	uptake
EX-co2(e)	4	secretion	[]
EX-gthrd(e)	4	[]	uptake
EX-h(e)	4	secretion	[]
EX-his-L(e)	4	[]	uptake
EX-i(e)	4	[]	uptake
EX-lpchol-hs(e)	4	[]	uptake
EX-tag-hs(e)	4	secretion	[]
EX-adp	3	[]	uptake
EX-atp(e)	3	[]	uptake
EX-gdp(e)	3	[]	uptake
EX-udp(e)	3	[]	uptake
EX-utp(e)	3	[]	uptake
EX-gtp(e)	2	[]	uptake
EX-nac(e)	2	[]	uptake
EX-pydx(e)	2	[]	uptake
EX-so4(e)	2	[]	uptake
EX-vitd3(e)	2	[]	uptake
EX-5hoxindoa[e]	1	secretion	[]
EX-pchol-hs(e)	1	[]	uptake
EX-s2l2n2m2masn(e)	1	[]	uptake
EX-strch1(e)	1	[]	uptake
EX-tymsf(e)	1	secretion	[]

5 Analyze the sets of essential genes

Use the function *analyzeSingleGeneDeletion* to predict and analyze the sets of essential genes across a set of models (*ResultsAllCellLines*, *samples*).

Define, if a heat map is generated (*heat* = 1). The output is saved to the specified location (*path*), and the results of the analysis are added to *ResultsAllCellLines*.
The output table (*genes*) consists of six columns specifying (1) the gene ID, (2) the category, (3) the number of models for which the gene is essential (growth ratio < 0.05), (4) the number of models for which the gene is not essential (growth ratio > 0.95), (5) the number of models in which the growth (ratio between wild-type and knock-out model) is reduced ($0.05 >$ growth ratio > 0.95), and (6) the number of models that do not have the gene.

Box 7

Input for matlab:

>> cutoff= 0.05;
>> [genes, ResultsAllCellLines, GeneKO_Matrix] = analyzeSingleGeneDeletion(ResultsAllCellLines, path, samples, cutoff, heat, OverViewResults);

6 Check reaction essentiality

Use the function *checkEffectRxnKO* to investigate, which reactions associated with a gene need to carry flux. Provide as input a (sub-)set of models (*samples_to_test*) and a set of genes (*genes_to_test*). Define a placeholder for an empty cell (*fill*).

Box 8

Input for matlab:

>> [FBA_Rxns_KO,ListResults] = checkEffectRxnKO(samples_to_test, fill, genes_to_test, samples, ResultsAllCellLines);

7 Make summary models

The function *makeSummaryModels* generates a union model (*unionModel*) and an intersect model (*intersectModel*). Additionally, it writes out the set of reactions (*diffRxns*) and exchange reactions (*diffExRxns*) that distinguish the union and the intersect model for further analysis. The function requires a generic *model* as a starting point.

Box 9
Input for matlab:

>> [unionModel, intersectModel, diffRxns, diffExRxns] = makeSummaryModels(ResultsAllCellLines, samples, model, mk_union, mk_intersect, mk_reactionDiff);

8 Predict strategies of metabolite production or consumption

Use the function *predictFluxSplits* to predict the production or consumption (*dir*) of metabolites of interest (e.g., *met2test* = atp;) for each model (*ResultsAllCellLines*) defined by *samples*. Define the objective function (*obj*) that should be used to generate the flux vector from which the reaction contributions for metabolite production/consumption are calculated. Define a full sized model (*model*), as the set of models can use distinct subsets of the reactions of the full model to produce or consume the metabolite of interest. The min norm option of the flux balance analysis (default *eucNorm* = 1e-6) is used to predict a flux distribution *BMall* as the basis for the calculation. This analysis requires a quadratic programming ('QP') solver.
In case of ATP production (*ATPprod*=1), additional statistics on the use of glycolysis, the citric acid cycle, and the oxidative phosphorylation are generated. Furthermore, an ATP yield (*ATPyield*) can be predicted,

which relates the total ATP production to a user-defined carbon source (default: *carbon_source* = 'EX_glc(e)'). The ATP yield can be used as a metric to distinguish metabolic phenotypes. Reported are the reactions carrying highest flux for production/consumption of the metabolite of interest (*maximum_contributing_rxn*, (*maximum_contributing_flux*) and details on (1) the maximum flux for producing the metabolite, (2) the sum of all fluxes producing the metabolite, and (3) the percent contribution of the maximum flux compared to the total flux. If the metabolite of interest is ATP, additional columns are added providing information on (4) the contribution of glycolysis ('PGK' and 'PYK'), (5) the contribution of ETC, (6) the combined contribution of glycolysis and ETC, (7) the contribution of citric acid cycle, and (8) the combined contribution of glycolysis, and ETC, and citric acid cycle to ATP production. Undesired reactions can be excluded from the calculation (*transportRxns*).

Box 10

Input for matlab:

```
>> eucNorm = 1e-6;
>> obj= 'biomass_reaction2';
>> [BMall, ResultsAllCellLines, metRsall, maximum_contributing_rxn, maximum_contributing_flux, ATPyield] = predictFluxSplits(model, obj, met2test, samples, ResultsAllCellLines, dir, eucNorm, transportRxns, ATPprod, carbon_source);
```

The output *metRsall* lists the relevant fluxes across the set of models. The detailed analyis is added to *ResultsAllCellLines*.

The analysis can be repeated for multiple metabolites and provided insight into how the samples produce important energy and redox metabolites by analyzing ATP, NADH, NADPH, and FADH2 (Table S 4).

Table 4. Reactions predicted to carry highest reaction fluxes for the production of important metabolites in 6 models. PGK = phosphoglycerate kinase, ATPS4m = ATP synthase, GAPD = glyceraldehyde-3-phosphate dehydrogenase, SUCD1m = succinate dehydrogenase, ICDHyrm = isocitrate dehydrogenase

samples	ATP	NADH	FADH2	NADPH
PC-3	ATPS4m	GAPD	SUCD1m	ICDHyrm
PC-3-2	ATPS4m	GAPD	SUCD1m	ICDHyrm
IGROV1	PGK	GAPD	SUCD1m	LALDD
IGROV1-2	PGK	GAPD	SUCD1m	LALDD
SNB-19	ATPS4m	GAPD	SUCD1m	ICDHyrm
SNB-19-2	ATPS4m	GAPD	SUCD1m	ICDHyrm

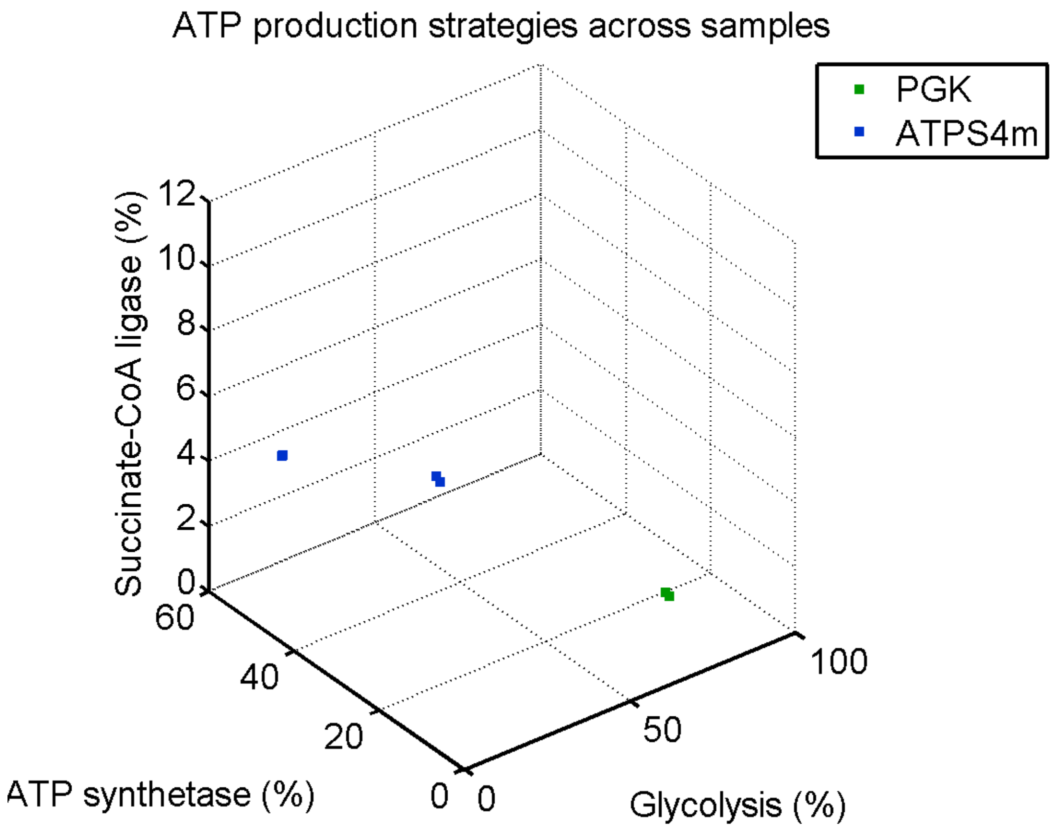

Figure 1. The phenotypes can be illustrated using the function *make3Dplot* after the calculation of the ATP production strategies using the function *predictFluxSplits*. Colors mark the major ATP producing reaction of the model.PGK= phosphoglycerate kinase or ATPS4m = ATP synthase.

9 Illustrate ATP production strategies on 3D plot

The function *make3Dplot* allows illustration of the results of the previous analysis. The colors specify different phenotypes.

Box 11

Input for matlab:

```
>> make3Dplot(PHs, maximum_contributing_flux_ATP, fonts, path, diff_view);
```

10 Phenotypic phase plane analysis (PPP)

Use the function *performPPP* to investigate the behavior of the models to variations in flux through a pair of exchange reactions. Box 12 illustrates the input for testing the robustness of the models to variation of glucose uptake & oxygen uptake, and glutamine secretion & oxygen uptake (*mets*). The range of flux values to be tested is defined by the number of steps and step size (*step_num*, and *step_size*). The direction of exchange is defined individually for each exchange (*direct*).

Box 12

Input for matlab:

```
>> mets ={'EX_glc(e)','EX_gln_L(e)';'EX_o2(e)','EX_o2(e)'};
>> step_size = [40,40; 40,40];
>> step_num = [20,20; 20,20];
>> direct = [-1,-1;-1,-1];
>> [ResultsAllCellLines] = performPPP(ResultsAllCellLines, mets, step_size,
samples, step_num, direct);
```

Subsequently, the function *illustrate_ppp* allows the illustration of the results as heatmap (Figure 2).

Box 13

Input for matlab:

```
>> illustrate_ppp(ResultsAllCellLines, mets, path, samples, label, fonts,
tol);
```

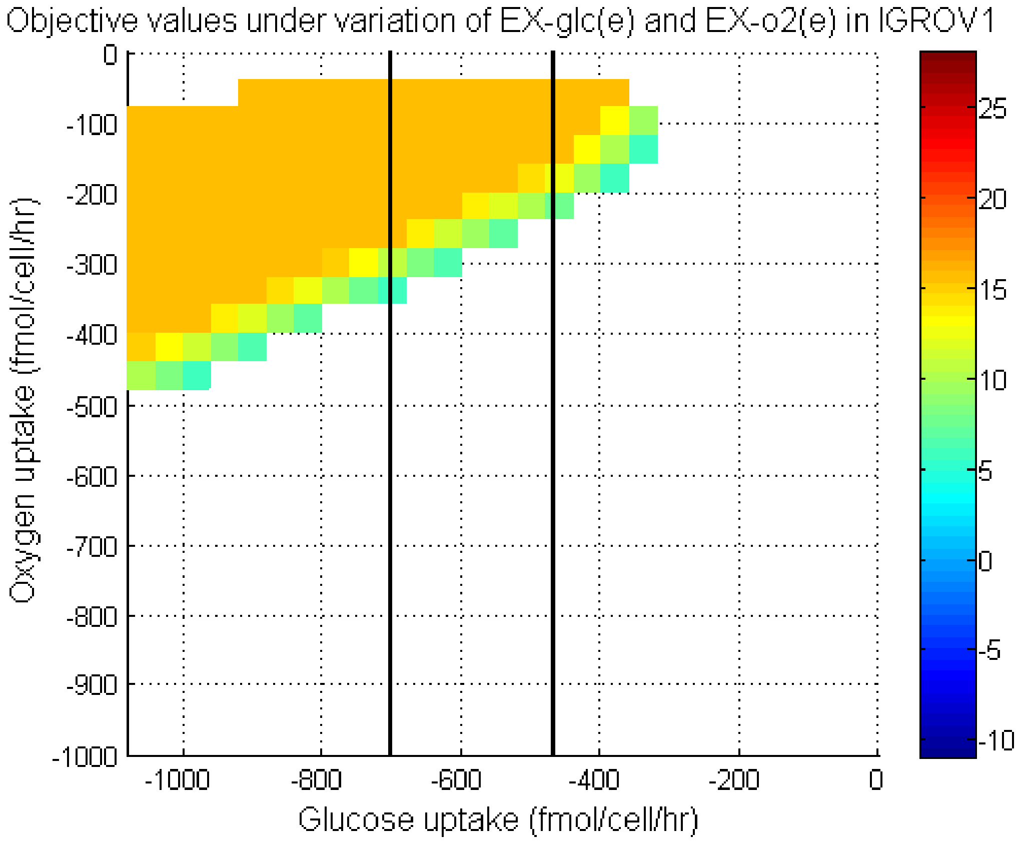

Figure 2. Heatmap of phase plane analysis.

References

1. Jain M, Nilsson R, Sharma S, Madhusudhan N, Kitami T, et al. (2012) Metabolite Profiling Identifies a Key Role for Glycine in Rapid Cancer Cell Proliferation. Science 336: 1040–1044.

2. Schellenberger J, Park J, Conrad T, Palsson B (2010) BiGG: a Biochemical Genetic and Genomic knowledgebase of large scale metabolic reconstructions. BMC Bioinformatics 11: 213.
